# Improved synthesis of [^18^F] fallypride and characterization of a Huntington’s disease mouse model, zQ175DN KI, using longitudinal PET imaging of D2/D3 receptors

**DOI:** 10.1186/s41181-019-0071-6

**Published:** 2019-08-05

**Authors:** Tuulia Huhtala, Pekka Poutiainen, Jussi Rytkönen, Kimmo Lehtimäki, Teija Parkkari, Iiris Kasanen, Anu J. Airaksinen, Teija Koivula, Patrick Sweeney, Outi Kontkanen, John Wityak, Celia Dominiquez, Larry C. Park

**Affiliations:** 1 Charles River Discovery Services, Microkatu 1, 70210 Kuopio, Finland; 2 Department of Neurobiology, A.I. Virtanen Institute for Molecular Medicine, University of Eastern Finland, 70211 Kuopio, Finland; 3 Department of Clinical Physiology and Nuclear Medicine, Kuopio University Hospital, 70210 Kuopio, Finland; 4 Department of Chemistry – Radiochemistry, University of Helsinki, 00014 Helsinki, Finland; 5 CHDI Management/CHDI Foundation, Los Angeles, CA USA

**Keywords:** Huntington’s disease, [^18^F] fallypride, PET, Autoradiography, Translational imaging

## Abstract

**Purpose:**

Dopamine receptors are involved in pathophysiology of neuropsychiatric diseases, including Huntington’s disease (HD). PET imaging of dopamine D2 receptors (D2R) in HD patients has demonstrated 40% decrease in D2R binding in striatum, and D2R could be a reliable quantitative target to monitor disease progression. A D2/3R antagonist, [^18^F] fallypride, is a high-affinity radioligand that has been clinically used to study receptor density and occupancy in neuropsychiatric disorders. Here we report an improved synthesis method for [^18^F]fallypride. In addition, high molar activity of the ligand has allowed us to apply PET imaging to characterize D2/D3 receptor density in striatum of the recently developed zQ175DN knock-in (KI) mouse model of HD.

**Methods:**

We longitudinally characterized in vivo [^18^F] fallypride -PET imaging of D2/D3 receptor densities in striatum of 9 and 12 month old wild type (WT) and heterozygous (HET) zQ175DN KI mouse. Furthermore, we verified the D2/D3 receptor density in striatum with [^3^H] fallypride autoradiography at 12 months of age.

**Results:**

We implemented an improved synthesis method for [^18^F] fallypride to yield high molar activity (MA, 298–360 GBq/μmol) and good reproducibility. In the HET zQ175DN KI mice, we observed a significant longitudinal decrease in binding potential (BP_ND_) (30.2%, *p* < 0.001, 9 months of age and 51.6%, p < 0.001, 12 months of age) compared to WT littermates. No mass effect was observed when the MA of [^18^F] fallypride was > 100 GBq/μmol at the time of injection. Furthermore, the decrease of D2/D3 receptor density in striatum in HET zQ175DN KI was consistent using [^3^H] fallypride autoradiography.

**Conclusions:**

We observed a significant decrease in D2/D3R receptor densities in the striatum of HET zQ175DN KI mice compared to WT mice at 9 and 12 months of age. These results are in line with clinical findings in HD patients, suggesting [^18^F] fallypride PET imaging has potential as a quantitative translational approach to monitor disease progression in preclinical studies.

## Introduction

Huntington’s disease (HD) is a devastating hereditary autosomal progressive neurodegenerative disorder. Clinical features of HD include chorea, cognitive dysfunction, and psychiatric symptoms that are caused by excessive repeat of CAG trinucleotide in the huntingtin gene (Htt) (MacDonald et al. 1993). Currently, symptomatic treatment is the only available option for HD (Vattakatuchery and Kurien 2013).

Alterations in the dopaminergic system play an important role in the pathophysiology of HD and other neuropsychiatric diseases such as Parkinson’s and Alzheimer’s disease, schizophrenia, and multiple sclerosis (Rangel-Barajas et al. 2015). Dopamine (DA) receptors are subdivided into D1-like and D2-like receptor families, and reduced density and activity of these receptors has been reported in the striatum of early-manifest HD patients (Niccolini et al. 2014). In another study about 40% reduction in binding of [^11^C]SCH23390 and [^11^C] raclopride to D1R and D2R, respectively, has been reported in HD patients (Ginovart et al. 1997). Reduction in radioligand binding was also significantly associated with the duration of symptoms, suggesting these receptors could be reliable quantitative targets to monitor disease progression (Niccolini et al. 2014; Ginovart et al. 1997).

[^11^C] Raclopride is a widely used tracer in D2R PET imaging (Niccolini et al. 2014; Ginovart et al. 1997) but, due to the short half-life of [^11^C] (20.3 min), [^18^F] based radioligands with a longer half-life (109.8 min) have been developed for use without close vicinity to a medical cyclotron. [^18^F] fallypride is a potent D2/D3 receptor antagonist, which has substantially higher affinity (33 pmol/L) to D2/D3 receptors compared to [^11^C] raclopride (26 nmol/L) in healthy humans, suggesting it could potentially be a useful agent to monitor neurodegenerative disease progression in HD (Mukherjee et al. 1995; Siessmeier et al. 2005; Elsinga et al. 2006).

Current HD mouse models include transgenic (TG) and knock-in (KI) models, of which KI model develops disease pathophysiology more slowly (Ehrnhoefer et al. 2009). The studied zQ175 delta neo KI (zQ175DN KI) mouse model is based on the established zQ175 KI mouse line that has been extensively characterized in molecular, behavioral, and imaging studies (Heikkinen et al. 2012; Menalled et al. 2012; Smith et al. 2014; Häggkvist et al. 2017). The zQ175 KI mouse model has an expanded CAG repeat in exon 1 of mouse *Htt* gene and already exhibits a HD-like phenotype when heterozygous (HET). However, the zQ175 KI allele has a neomycin resistance cassette in the *Htt* gene locus, which has been removed in the zQ175DN KI strain. This new strain has not been as well characterized as its predecessor, but recently it was reported that onset of HD-like symptoms appear earlier and disease pathology develops more rapidly in zQ175DN KI mice than in zQ175 KI mice (Heikkinen et al. 2017).

Applying [^18^F] fallypride to mouse PET imaging could significantly improve throughput compared to [^11^C] raclopride, with some potential caveats. For example, in rodent D2R/D3R imaging, low molar activity may affect the results as too high of an injected mass may cause saturation of the receptors (Alexoff et al. 2003). Therefore, a highly optimized synthesis method is crucial for successful PET imaging in rodents. Here we present an improved synthesis method for [^18^F]fallypride. We also characterized the zQ175DN KI mouse in vivo using [^18^F] fallypride PET imaging and ex vivo [^3^H] fallypride autoradiography, following the progression of phenotype with PET scans at the age of 9–10 months and again at 12–13 months. Finally, we applied autoradiography to the measure of D2/D3 receptor density in the striatum.

## Materials and methods

### Chemicals and instrumentation

All chemicals and solvents were purchased from Sigma-Aldrich (Milwaukee, WI, USA) and used without further purification. Carbonated QMA light and C18 light cartridges were purchased from Waters (Milford, MA, USA), sterile Millex-GV filters (0.22 μm) were purchased from Millipore (Billerica, MA, USA), absolute ethanol was purchased from Altia (Finland) and sterile water and saline were purchased from Braun. Sterile 15 mL vials were purchased from Comecer (Italia).

Semi-preparative high-performance liquid chromatography (HPLC) was conducted in the GE TRACERlab™ FX2-N under the following HPLC conditions: Phenomenex Luna (C2) C18 column, 250 × 10 mm, 5 μm; ACN:Water 40/60 + 0.1 M ammoniumformate; 4 mL/min. Analytical HPLC was performed using an Agilent 1260 HPLC system, which includes a quaternary gradient pump, a variable wavelength detector, and a radioactivity-HPLC-flow detector. Analytical HPLC was performed under the following conditions: Phenomenex Kinetex C18 4.6 × 50 mm, 5 μm, ACN:Water 37:63 + 0.1% TFA, 2 mL/min.

The elution efficiency of [^18^F] fluoride ion from a carbonated QMA light cartridge was evaluated with K_2_CO_3_ and Kryptofix 2.2.2. Acetonitrile (ACN)/water/theoxylalcohol was used as the eluent for eluting trapped [^18^F] fluoride ion. The results (data not shown) indicated that 0.8 mg K2CO3 and 9.1 mg K222 were able to eluate the [^18^F] fluoride ion from Waters QMA light cartridge. Reducing the amount of K_2_CO_3_ to 0.5 mg decreased the eluation efficacy.

### Synthesis of [^18^F]fallypride

**Scheme 1:**
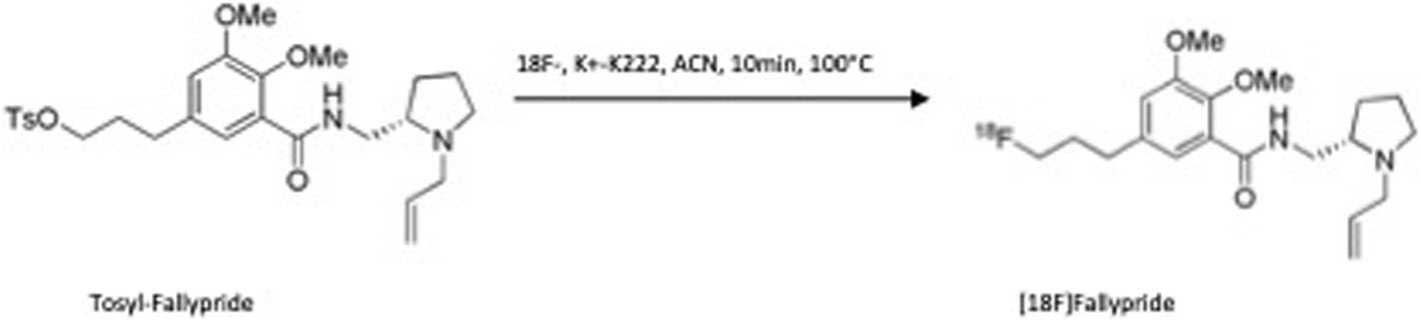
The reaction scheme for the synthesis of [^18^F]fallypride.

When imaging rodents, especially mice with limited neuroreceptors, high molar activity radioligands should be used to enable reliable quantification of differenes between individuals. For this purpose, improved synthesis method was established. Overview of the reaction is shown in Scheme 1. The optimized radiosynthesis of [^18^F] fallypride was completed via the following procedure: 1) [^18^F] fluorination, 2) HPLC purification, and 3) formulation. The synthesis of [^18^F] fallypride consisted of seven (7) reagent vials on the FX2-N module. Vial 1 was used for the elution. Vial 3 was used for precursor; vials 4–5 for quenching reagent; vials 12–13 for the formulation of purified [^18^F]fallypride; and vial 14 for the washing of C18 cartridge after trapping the final product from HPLC. Vial 1 was added with fresh 9.1 mg K222, 0.8 mg K2CO3 in 1.1 mL ACN, thexyl-alcohol, water (8:2:1); Vial 3 was added with the tosylate precursor (0.8 mg) in ACN (0.8 mL); Vial 4 was added with 30:70 ACN:water solvent (2 mL); Vial 5 was added with HPLC solvent (1 mL); Vial 12 was added with saline (7 mL); Vial 13 was added with EtOH (0.7 mL); Vial 14 was added with sterile water (10 mL); and an HPLC dilution flask was added with water (30 mL).

Typically, 37 GBq (1 Ci) [^18^F] fluoride in 2.5 mL of water was passed through the QMA cartridge and dried with helium flow. [^18^F] Fluoride was eluted from the cartridge into reactor 1 (R1) with the eluent in Vial 1, and azeotropically dried at 55 °C with N_2_ flow for 3 min and at 75 °C for 3 min under N_2_/vacuum. The activity was further dried using a vacuum for 5 min. The [^18^F] fluoride drying cycle took about 11 min. The tosylate precursor in Vial 3 was added to the dried activity. The resulting solution was stirred at 100 °C for 10 min, and cooled to 45 °C. The reaction mixture was diluted with 30:70 ACN:water (Vial 4), and transferred to an intermediate tube. Reactor 1 was rinsed with another portion of ACN:water (Vial 5) and the solution was also transferred to an intermediate tube. The solution in Tube 2 was thoroughly mixed by bubbling N_2_ for 15 s and injected into the HPLC for purification. The labeled product was eluted at about 16 min. The product peak was collected in the dilution flask containing 30 mL of water, and passed through a Waters C18 light cartridge (pre-conditioned with 5 mL of water, 5 mL of ethanol, and 5 mL of water). The trapped intermediate was rinsed with 10 mL of water (Vial 14), and eluted with 0.7 mL of absolute ethanol (Vial 13) after which the C18 cartridge was rinsed with 7 mL of sterile saline. The content was transferred into the final product vial with an in-line sterile filter.

The chemical identity, MA, and radiochemical purity (RCP) of the [^18^F] fallypride was checked by analytical HPLC or radio-TLC. The stability of the product was verified until next day (18 h, HPLC).

### Animals

All animal experiments were carried out according to the National Institutes of Health (NIH, U.S.) guidelines for the care and use of laboratory animals and approved by the National Animal Experiment Board (Finland). The animal facility has been accredited by the Association for Assessment and Accreditation of Laboratory Animal Care International (AAALAC). All procedures performed in studies involving animals were in accordance with the ethical standards of the institution or practice at which the studies were conducted.

Eight-month-old male HET zQ175DN KI mice and their WT littermates were purchased from Jackson Laboratories. In order to prevent fighting between male mice housed together, the studied male mice were housed separately in individually ventilated cages accompanied with a female for the entire study duration. The mice had access to chow and water ad libitum. Prior to accepting the mice to the study, they were screened for abnormal liver function with Diazyme Total Bile Acids Assay (Diazyme Europe GmbH, Dresden, Germany) from plasma samples.

### PET imaging and image analysis

The mice were anesthetized using isoflurane (4–5% induction, 1.5–2% maintenance) and cannulated in the lateral tail vein. The cannula was filled with heparinized saline (100 IU/mL) to prevent blood coagulating in the cannula. Three mice were cannulated simultaneously and transferred into a scanner (BioPET/CT, Sedecal, Spain) equipped with a heated small animal bed with a holder for three mice (Minerve Veterinary Equipment, France). The rectal temperature and breathing rate of the animals were monitored during the scan and adjusted accordingly to 37 °C and 70–90 bpm. A bolus of [^18^F] fallypride (150 μL; mean 9.7 MBq; MA > 100 GBq/μmol) was administrated 30 s after the start of the dynamic PET scan. List mode data was acquired for 90.5 min. After each PET scan a CT scan was performed (140 μA, 45 kV) for information on individual animal orientation and attenuation map for image reconstruction. The acquired sinograms were reconstructed with 3D OSEM (1 iteration, 25 subsets) applying attenuation correction into 30 time frames (1 × 30 s; 4 × 10 s; 4 × 20 s; 4 × 60 s; 6 × 180 s; 11 × 360 s).

The dynamic PET images were analyzed with PMOD software v3.7 (PMOD Technologies Ltd., Zürich, Switzerland). The PET images were co-registered with an in-house reconstructed MRI template created from the image MRI of average 12-month-old WT and HET zQ175 KI mice (*n* = 10/genotype). Volumes of interest (VOIs) of striatum and cerebellum of the template were used. The VOI of cerebellum had a reduced size to prevent spillover signal from defluorinated [^18^F] fluoride uoride accumulating into bones of the skull. The dynamic PET data was fitted to a simplified reference tissue model (Lammertsma and Hume 1996) and the nondisplaceable binding potential (BP_ND_) of [^18^F] fallypride was measured in the striatum. Average standardized uptake value (SUV) images from each group and time activity curves (TAC) from striatum and cerebellum were calculated.

### Autoradiography

After the PET imaging at the age of 12 months the mice were decapitated and their brains were quickly frozen in dry ice-cooled isopentane (− 55° – -45 °C) and stored at − 80 °C until sectioning. The brains were cryosectioned into 20 μm thick slices and mounted on superfrost glass slides (Thermo scientific) and stored at − 80 °C until staining. The slides were quickly thawed under a stream of air and incubated with [^3^H] fallypride (80.6 Ci/mmol, 1470, RC Tritec AG) in assay buffer (50 mM Tris-HCl, 1 mM EDTA, 100 mM NaCl, 5 mM MgCl_2_, pH 7.4) for 60 min at room temperature. To determine B_max_ for D2/D3R the slides were incubated with the following ligand concentrations: 0.01, 0.05, 0.1, 0.2, 0.5, 1.0, 1.5, 2.0, 5.0, and 10.0 nM. Non-specific binding was measured by competing the radioligand against 10 μM of unlabeled fallypride. After the incubation, the slides were washed 2 × 5 min in 50 mM Tris-HCl, 5 mM MgCl_2_ pH 7.4, and milliQ water. Digital autoradiography images were acquired with BetaImager TRacer (BiospaceLab, France) with 6 h exposure time. ROIs were manually drawn on striatum using M3 Vision software (v1.0.6.1035, BiospaceLab, France) and specific binding was calculated as the difference of total and non-spesific binding. Non-linear regression analysis (GraphPad Prism v7.00, GraphPad Software Inc., La Jolla, CA, USA) was used determine B_max_ of D2/D3 receptors.

### Statistical analysis

Mean values ± SD are presented for all results. Comparison between groups has been done with unpaired student’s t-test unless otherwise stated. All statistics and graphs have been calculated with GraphPad Prism.

## Results

The radiosynthesis of [^18^F] fallypride was performed with good yield, and its molar activity was reproducible. The synthesis route is presened in Sheme 1. In addition, radiochemical purity was high (> 99%). The overall synthesis yield was approximately 55% (non decay corrected) with total synthesis time of 58 min. The stability of the product up to 18 h was verified using radio-HPLC. Our synthesis method didn’t produce any carbonated impurities that have previously been observed during [^18^F] fallypride synthesis. This could be observed from the radio-HPLC chromatograms. The molar activity (MA) of [^18^F] fallypride was 300–550 GBq/μmol, which allows two subsequent PET scans, as opposed to one scan from the typical synthesis batch with MA < 100 GBq/μmol at the time of injection.

**Fig. 1:**
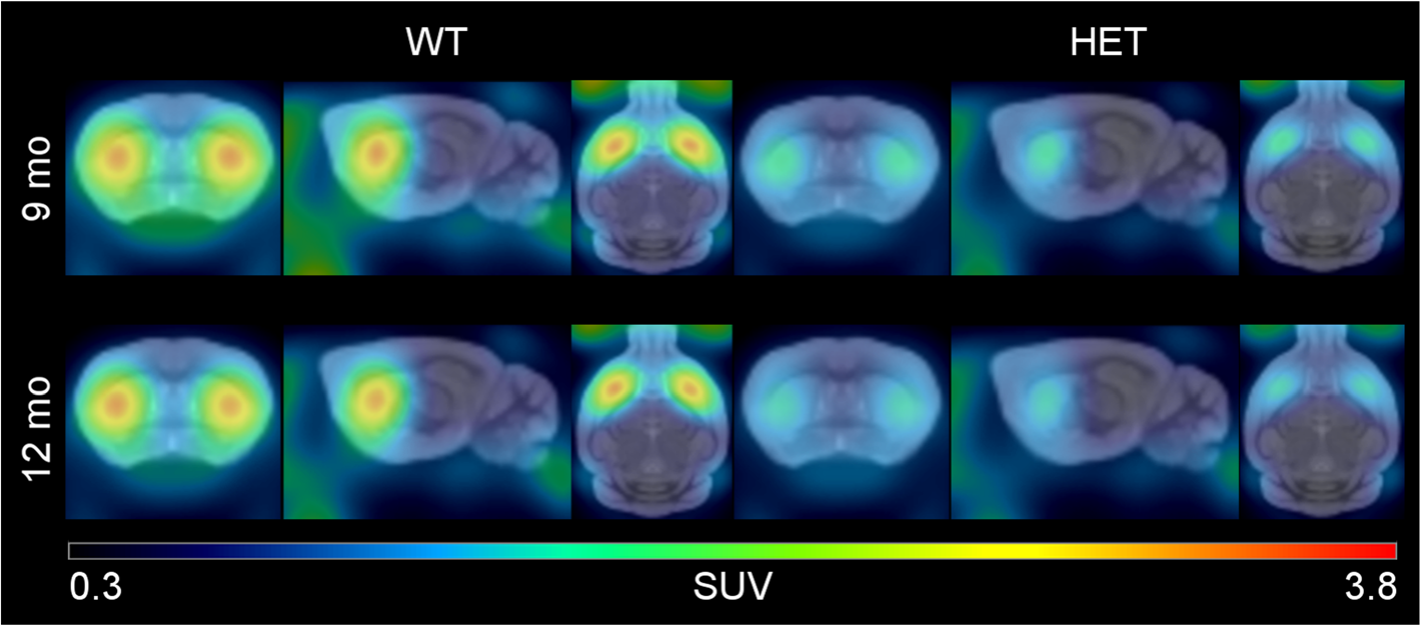
Averaged PET images from 45 to 90 min post injection showed higher uptake in the striatum of WT mice compared to HET both at 9 and 12 months. The PET images have been co-registered with in-house generated MRI template for visualization

**Fig. 2:**
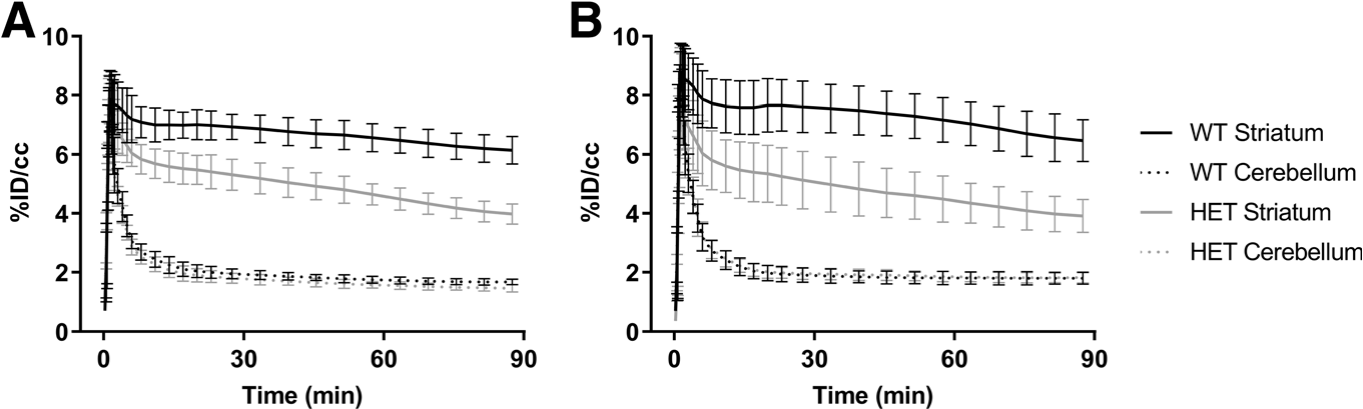
Time activity curves of [^18^F] fallypride in striatum and cerebellum (reference region) at the age of 9 months (**a**) and 12 months (**b**). Data is presented as % of injected dose/cubic centimeter of tissue (%ID/cc) ± SD

**Fig. 3:**
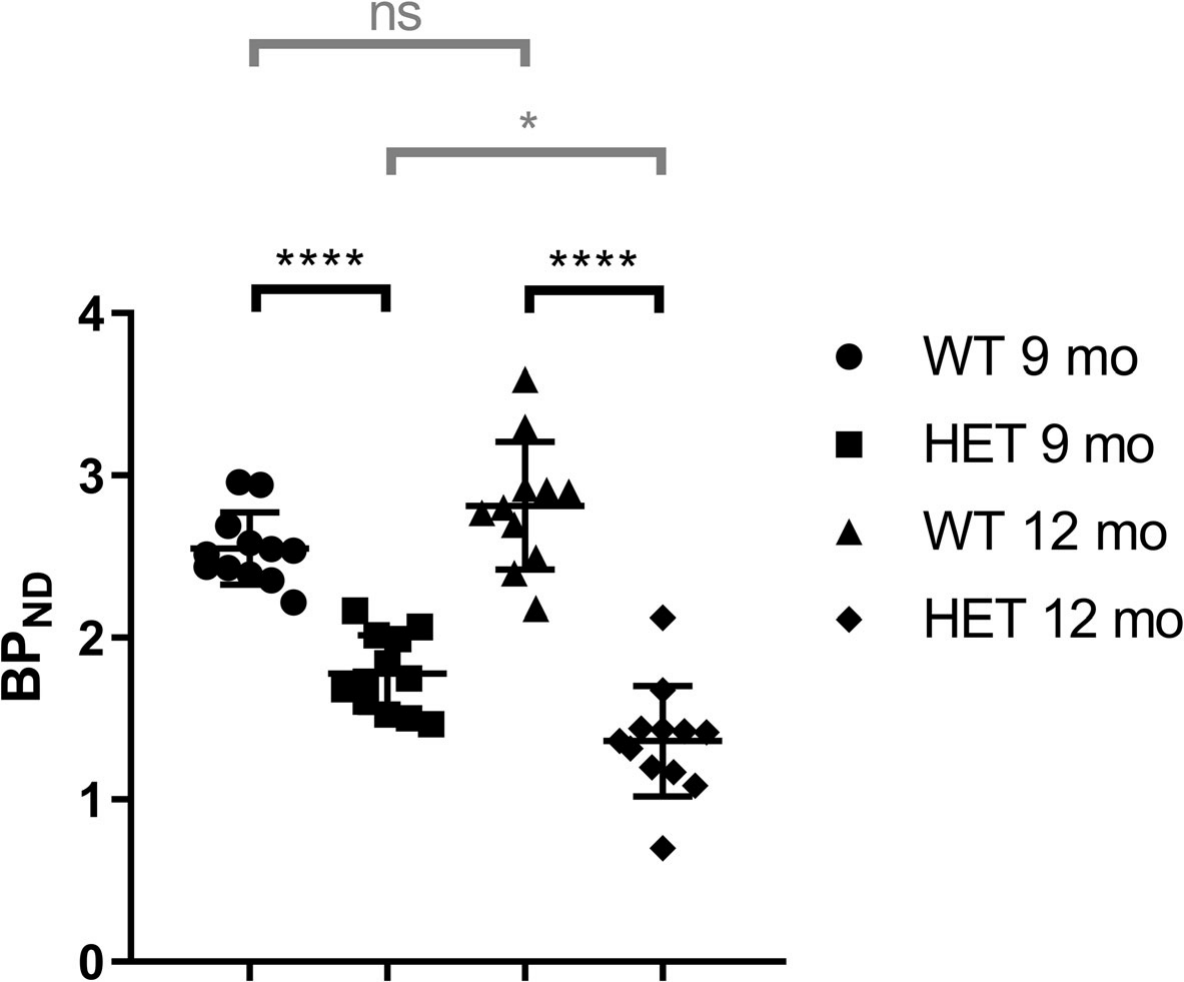
Individual and mean ± SD binding potential of [^18^F] fallypride in WT and HET zQ175DN KI mice. Statistical analysis using paired two-tailed T-test (gray line) and unpaired two-tailed T-test (black line, ns = nonsignificant, *** *p* = 0.005, **** *p* < 0.0001)

Average PET images showed a high accumulation of [^18^F] fallypride to the striatum, whereas no sign of tracer was seen in cerebellum during 45–90 min post injection (Fig. 1). The binding of [^18^F] fallypride in striatum of HET zQ175DN KI mice was significantly reduced compared to their WT littermates. [^18^F] fallypride had slow kinetics in the striatum, which could be seen from the TACs (Fig. 2). When individual binding potentials (BP) including mean value and SD were calculated using SRTM, the striatum of zQ175DN HET mice showed a 30.2% decrease in BP_ND_ (*p* < 0.0001) 9 mo and 51.6% decrease (p < 0.0001) at 12 mo when compared to those in WTs (Fig. 3). BP_ND_ of WT showed a 10.1% increase from 9 to 12 mo, which was not statistically significant. On the other hand, 23.6% decrease (*p* < 0.05) was seen from 9 mo to 12 mo of zQ175KI mice.

**Fig. 4:**
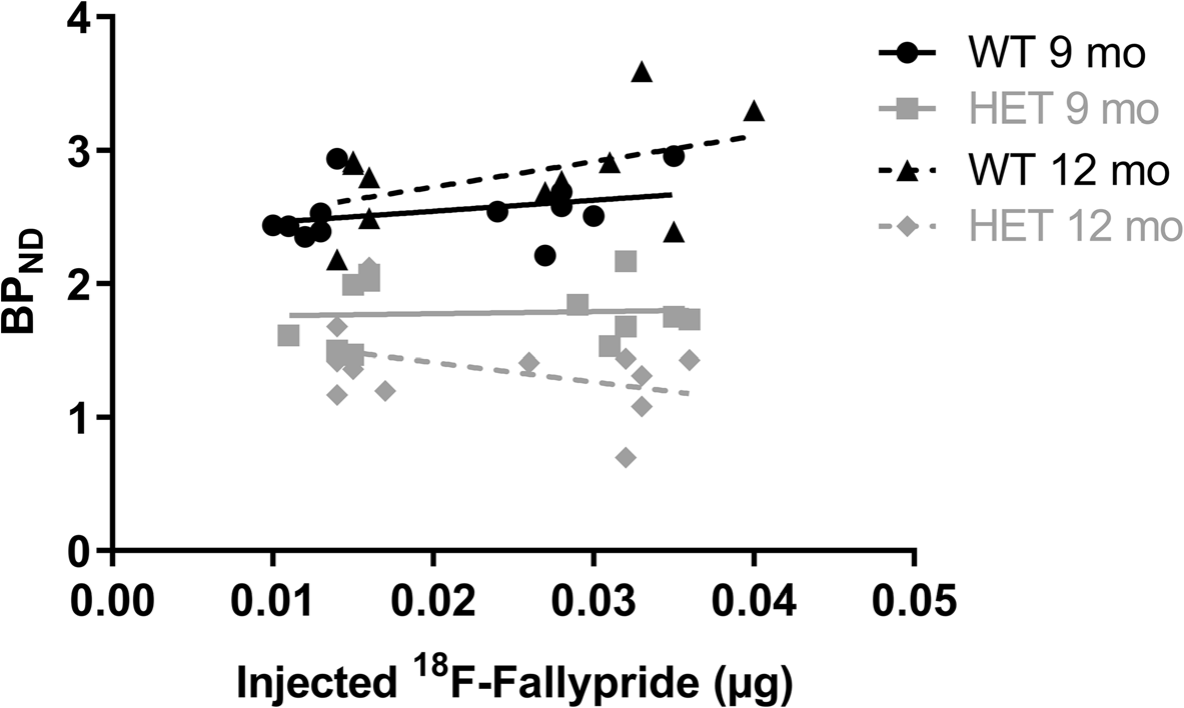
No mass effect to BP_ND_ of [^18^F] fallypride was observed when using high specific activity (MA > 100 GBq/μmol) at the time of injection. Line represents linear regression of the population

As 3 mice were scanned simultaneously, good throughput of 6 mice/synthesis was achieved. No mass effect and receptor saturation was observed with MA > 100 GBq/μmol at the time of injection (Fig. 4) as no correlation for injected mass and BP_ND_ was observed (Pearson correlation, Table 1).

**Table 1:** Pearson correlation of injected mass of [^18^F] fallypride and BP_ND_. No significant correlation was observed

Genotype	Pearson r	R^2^	P (two-tailed)	n
WT 9 mo	0.334	0.111	0.289	12
HET 9 mo	0.064	0.004	0.844	12
WT 12 mo	0.462	0.213	0.153	11
HET 19 mo	−0.393	0.155	0.206	12

**Fig. 5:**
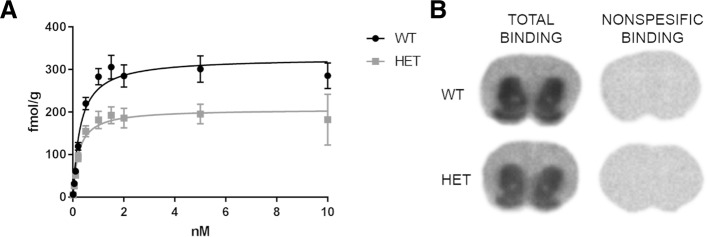
**a** Specific binding of [^3^H] fallypride in striatum of WT and HET zQ175DN KI mice (*n* = 12/genotype). **b** Representative sections of total and nonspecific binding in WT and HET mice brain after [^3^H] fallypride staining (10 nM)

In ex vivo autoradiography, 35% reduction in B_max_ of [^3^H] fallypride in striatum of HET zQ175DN KI mice was observed in comparison to WT (Extra sum-of-squares F-test, *P* < 0.0001). The B_max_ values were 327.3 ± 5.4 fmol/g and 206.8 ± 4.6 fmol/g for WT and HET, respectively. The majority of the [^3^H] fallypride binding occurred in striatum and nucleus accumbens. Non-specific binding of radioligand was minimal (Fig. 5).

## Discussion

Low abundance of target receptors, accurate tracer kinetics modeling, pharmacological effects, and toxicity of the imaging probes are examples of why high molecular activity (MA) is desired in PET imaging. Low MA increases the injected mass of the tracer, which can have unknown pharmacological effects or even saturate the receptor, biasing the binding potential.

Low MA can affect the results of rodent D2R/D3R imaging when the injected mass is too high, which causes saturation of the receptors (Alexoff et al. 2003). In the current study, we observed no mass effect with the animals dosed with an average of 9.7 MBq with MA > 100 GBq/μmol of [^18^F]fallypride. This is in line with previous studies where no mass effect was observed with female BALB/c mice injected with 6 MBq of [^18^F] fallypride (MA > 50 GBq/μmol) (Rominger et al. 2010).

Previously, optimization of [^18^F] fallypride synthesis had been done with microfluidic reactors up to a MA of 730 MBq/μmol (Javed et al. 2014); however, batch size using this process is too small for clinical use and scale-up is difficult. Enhanced reaction kinetics can potentially increase the fluorination kinetics of a [^18^F] fluoride ion in a reactor during the labeling. Usually the major contaminant of [^19^F] is found from the target water, target material, tubings, solvents, and anion exchange resins and reactor wall. The amount of [^19^F] is much higher compared to [^18^F], indicating the MA should be directly proportional to the amount of [^18^F] at the start of labeling. However, this is not always the case and attempts to increase the MA of [^18^F] fallypride have often failed.

A moderate radiochemical yield (RCY 20–40%) for [^18^F] fallypride has been reported (Javed et al. 2014). Long labeling time (15–30 min) and high temperatures (over 150 degree Celsius) are problematic since they usually demand lengthy HPLC purification steps to avoid mass peaks, especially when using over 2 mg of a precursor. The use of high base concentrations in the [^18^F] labeling step is likely to be the main reason for various side products and consequently low radiochemical yield, particularly in the case of base-sensitive tosyl-fallypride. Here we synthetized [^18^F] fallypride by labeling tosyl-fallypride (tosylate precursor) with [^18^F] fluoride ion using a phase-transfer catalyst in GE’s Tracerlab FX2-N synthesis module. Using low concentrations of K_2_CO_3_ is desirable since high concentrations may lead to side products. In order to keep the amount of K_2_CO_3_ reasonable, we used K2.2.2/K_2_CO_3_ (9.1 mg/0.8 mg). One hypothesis for improved reaction kinetics and increased nucleophilicity of the [^18^F] fluoride ion is addition of thexyl alcohol into the eluting solution. The elution was carried out in acetonitrile/theoxylalcohol/water solution (8:2:1, 1.1 mL) and the drying of the [^18^F] fluoride was carried out in a single step without adding any extra acetonitrile. In summary, by using a low amount of K_2_CO_3_ and with addition of thexyl alcohol, the RCY yield was 55% (non decay corrected) with a MA of 300–550 GBq/umol. The reaction can be carried out in 16 mL reactor in ACN 10 min using a regular heater at 100 °C.

In previous small-animal PET imaging with [^11^C] raclopride using the zQ175 KI mouse strain, the BP_ND_ difference to WT animals was 40% and 44% at the ages of 6 months and 9 months, respectively (Häggkvist et al. 2017). Our results are in line with these findings, although the results are not fully comparable as the mouse lines are slightly different, and chemical structures of the tracers are not identical. This is encouraging as both [^11^C] raclopride and [^18^F] fallypride are well known clinical PET ligands for D2/D3 antagonism. For the first time, improved [^18^F] fallypride ligand synthesis enabled a proper characterization of the D2/D3 binding potential changes in HD model using [^18^F] radiotracer. With improved MA, [^18^F] fallypride is unique in that it is the only currently available PET radiotracer that can simultaneously provide quantitative measures of D2/D3 receptor binding in the striatum and possibly extrastriatal brain regions in the same scanning session in HD models, which remains to be tested.

## Conclusions

Succesful preclinical PET imaging of neurologically important receptors requires delicate radiosynthesis outcome to enable quantification of differences between individulas in reliable manner. To achieve that, applying improved [^18^F] fallypride synthesis method in longitudinal PET imaging, we were able to characterize a longtidinal D2/D3 receptor level change in striatum of HET zQ175DN KI mice compared to WT mice. This significant decline of D2/D3 receptor binding potentials over time in HD mouse striatum was also confirmed by ex vivo autoradiography. As these pathophysiological changes are in line with clinical findings in HD patients, [^18^F] fallypride PET imaging can be considered as a quantitative therapeutic approach in longitudinal preclinical studies.

## Data Availability

NA, relevant results and data included in the publication.

## Abbreviations

AAALAC: Assessment and Accreditation of Laboratory Animal Care International
ACN: Acetonitrile
BPND: Nondisplaceable binding potential
CT: Computed tomography
D2R: Dopamine D2 receptors
DA: Dopamine
HD: Huntington’s disease
HET: Heterozygous
HPLC: High-performance liquid chromatography
KI: Knock-in
MA: Molar activity (Bq/mol)
MRI: Magnetic Resonance Imaging
NIH: National Institutes of Health
OSEM: Ordered subsets expectation-maximization
PET: Positron emission tomography
RCP: Radiochemical purity
RCY: Radiochemical yield
ROI: Region of interest
SRTM: Simplified reference tissue model
SUV: Standardized uptake value
TAC: Time activity curve
TFA: Trifluoroacetic acid
TG: Transgenic
TLC: Thin-layer chromatography
VOI: Volume of interest
WT: Wild type
